# Efficacy of Ceftobiprole and Daptomycin at Bone Concentrations Against Methicillin-Resistant *Staphylococcus aureus* Biofilm: Results of a Dynamic In Vitro PK/PD Model

**DOI:** 10.3390/antibiotics14040386

**Published:** 2025-04-05

**Authors:** Mikel Mancheño-Losa, María Ángeles Meléndez-Carmona, Carlos Lumbreras, Jaime Lora-Tamayo

**Affiliations:** 1 Department of Internal Medicine, Hospital Universitario 12 de Octubre, Instituto de Investigación “i + 12” del Hospital 12 de Octubre, 28041 Madrid, Spain; 2 Department of Clinical Microbiology, Hospital Universitario 12 de Octubre, Instituto de Investigación “i + 12” del Hospital 12 de Octubre, 28041 Madrid, Spain; 3 CIBER de Enfermedades Infecciosas CIBERINFEC, Instituto de Salud Carlos III, 28029 Madrid, Spain; 4 Department of Medicine, School of Medicine, Complutense University of Madrid, 28040 Madrid, Spain

**Keywords:** biofilm, MRSA, prosthetic joint infection, ceftobiprole, daptomycin

## Abstract

**Background**: The presence of biofilms and low antimicrobial concentrations in bone tissue make prosthetic joint infections (PJI) difficult to treat. Ceftobiprole (CTO) has a potential role in MRSA PJI. This study evaluated the efficacy of ceftobiprole and daptomycin (DAP) alone and in combination against MRSA biofilms at expected bone tissue concentrations. We assessed whether CTO-DAP outperformed DAP combined with a non-anti-MRSA beta-lactam (cefazolin [CZO]). **Methods**: A dynamic in vitro PK/PD biofilm model (CDC biofilm reactor) was used to simulate concentrations expected in cortical bone at a standard dosing of DAP (10 mg/kg/24 h), CTO (500 mg/8 h), and CZO (2 g/8 h), and assess performance against a 48-h MRSA biofilm from two clinical isolates that cause PJI (MRSA-1811 and MRSA-1733). Time–kill curves using the log change method (Δlog_10_ CFU/cm^2^) assessed antimicrobial efficacy over 56 h. Resistance emergence was monitored. **Results:** Although both monotherapies were active, neither reached bactericidal levels nor was one superior to the other (Δlog_10_ CFU/cm^2^ CTO vs. DAP: −1.44 ± 0.25 vs. −1.50 ± 0.01 [*p* = 0.686] and −1.55 ± 0.74 vs. −0.56 ± 0.36 [*p* = 0.108] for MRSA-1811 and MRSA-1733, respectively). Only in the MRSA-1811 isolate did the CTO-DAP combination improve the activity of each monotherapy, without achieving a synergistic effect (Δlog_10_ CFU/cm^2^: CTO-DAP −2.087 ± 0.048 vs. CTO −1.436 ± 0.249 [*p* = 0.013] and vs. DAP −1.503 ± 0.011 [*p* = 0.006]). No combination therapy (CTO-DAP vs. DAP-CZO) outperformed the other in either strain. No resistant bacterial subpopulations appeared with any antibiotic regimen. **Conclusions:** At clinically relevant concentrations, ceftobiprole and daptomycin showed similar activity against MRSA biofilms. The CTO-DAP combination showed comparable efficacy to DAP-CZO.

## 1. Introduction

Biofilm-associated infections are considered difficult to treat due to several bacterial mechanisms, such as phenotypic antimicrobial tolerance and persistence, that render antibiotics ineffective [1]. In the case of periprosthetic joint infections (PJIs), additional difficulties include poor antibiotic penetration into the bone and low antimicrobial concentrations at the site of infection [2]. *Staphylococcus aureus* is a common cause of PJI and is associated with a poor prognosis [3], especially when methicillin-resistant (MRSA) [4].

In this setting, daptomycin has been proposed as an effective treatment option during the early postoperative period, when the bacterial load remains high, surgical drains are still in place, and the wound has not yet healed [5]. Under these conditions, the elevated risk of selecting resistant mutants precludes the use of other biofilm-active agents, such as rifampicin [2]. Daptomycin in combination with beta-lactams prevents the emergence of bacterial resistance and has been shown to have synergistic activity against MRSA [6], including biofilm-embedded bacteria [7]. This effect is the result of a reduction in the positive bacterial surface charge induced by beta-lactams, leading to an increased affinity for the cationic peptide daptomycin [6], even when the beta-lactam used has no intrinsic activity against the strain. However, little is known about whether daptomycin plus a beta-lactam with intrinsic activity against MRSA has better anti-biofilm activity.

Ceftobiprole is a broad-spectrum cephalosporin that has demonstrated anti-MRSA biofilm activity in vitro [8] and in animal models of osteomyelitis and foreign body infection [9,10]. In combination with daptomycin, it has shown in vitro synergy in a planktonic infection model [11]. However, the efficacy of this combination against biofilm-embedded MRSA needs to be investigated. It is also important to challenge antibiotics at realistic concentrations found in bone tissue. Our aim, using a validated pharmacokinetic/pharmacodynamic (PK/PD) model, was to evaluate the activity of ceftobiprole as monotherapy and in combination with daptomycin against MRSA biofilms at expected bone antibiotic concentrations.

## 2. Results

Both strains were susceptible to daptomycin and ceftobiprole. As expected, they were not susceptible to cefazolin. The MICs and MBECs are shown in Table 1. None of the antibiotics showed an MBEC value achievable at standard clinical dosing.

### 2.1. Pharmacokinetics

The observed PK values are summarized in Table 2 and Supplementary Figure S1. Overall, PK validation of the model was robust, with all parameters within 10% of the target values.

### 2.2. Biofilm Time–Kill Analyses

**Figure 1:**
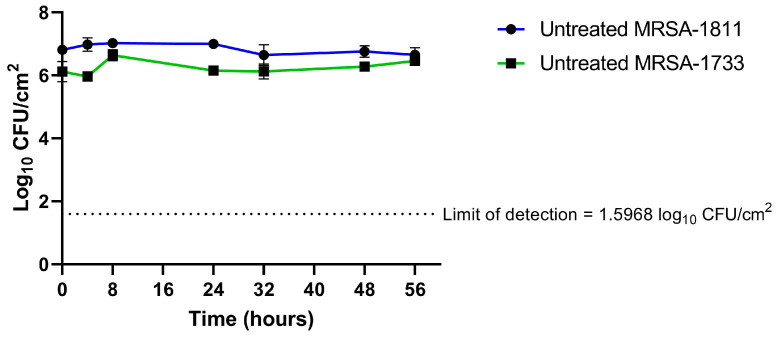
Growth controls for biofilm-embedded bacteria. Time on the *x* axis begins after the 48-h conditioning phase. Data are presented as means ± SD.

The initial inoculum (t = 0 h) of the MRSA-1811 strain was significantly higher than MRSA-1733 (6.92 ± 0.12 log_10_ CFU/cm^2^ vs. 5.76 ± 0.38 log_10_ CFU/cm^2^, *p* = 0.0002). Both strains showed stable bacterial densities in the absence of antibiotics (Figure 1).

**Figure 2:**
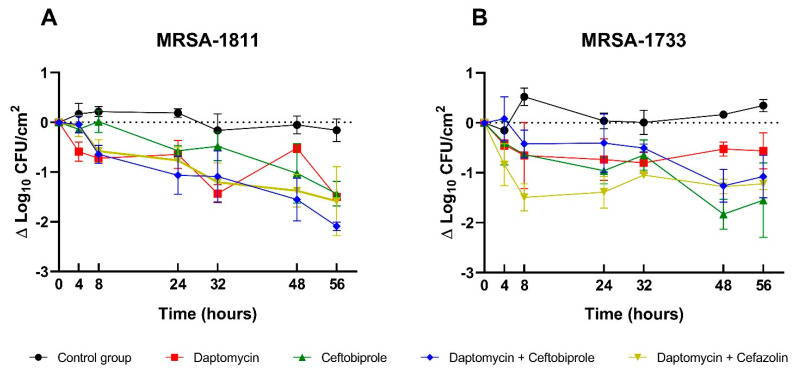
Time–kill curves of ceftobiprole, daptomycin, and combination regimens against two biofilm-embedded clinical strains of MRSA: MRSA-1811 (**A**) and MRSA-1733 (**B**). Data are presented as means ± SD.

The activity of the antimicrobial regimens is shown in Figure 2. For the monotherapies, both daptomycin and ceftobiprole showed significant bacterial count reductions compared to controls, but neither achieved bactericidal activity against any of the strains. The activities of both monotherapies were alike. Although not statistically significant, at 56 h, ceftobiprole alone was the most effective treatment against the MRSA-1733 strain, showing a one-log reduction compared to daptomycin (Δlog_10_ CFU/cm^2^ = −1.55 ± 0.74 vs. −0.56 ± 0.36, *p* = 0.108).

Ceftobiprole demonstrated comparable activity between the two strains, with minimal inter-strain variability (Δlog_10_ CFU/cm^2^ = −1.547 ± 0.743 vs. −1.436 ± 0.249, *p* = 0.818). In contrast, daptomycin was significantly more active against the MRSA-1811 strain than against MRSA-1733 (Δlog_10_ CFU/cm^2^ = −1.503 ± 0.011 vs. −0.562 ± 0.360, *p* = 0.045).

Against the MRSA-1811 strain, the combination of cefazolin plus daptomycin showed no better activity than daptomycin alone. However, the combination of ceftobiprole plus daptomycin (Δlog_10_ CFU/cm^2^ = −2.087 ± 0.048) was more active than either monotherapy (CTO −1.436 ± 0.249, *p* = 0.013; DAP −1.503 ± 0.011, *p* = 0.006), although it did not achieve additive or synergistic effects. Conversely, no such improvement was observed for the MRSA-1733 strain, but the combination of daptomycin plus cefazolin was more active than daptomycin alone (Δlog_10_ CFU/cm^2^ = −0.562 ± 0.360 vs. −1.222 ± 0.118, *p* = 0.039).

No combination therapy was superior to another in either strain. Ceftobiprole in combination with daptomycin was the most active regimen against the MRSA-1811 strain, showing a 0.5-log_10_ greater reduction compared to daptomycin plus cefazolin (Δlog_10_ CFU/cm^2^ = −2.087 ± 0.048 vs. −1.586 ± 0.695, *p* = 0.283). Furthermore, this regimen was significantly more active against the MRSA-1811 strain than against MRSA-1733 (Δlog_10_ CFU/cm^2^ = −2.087 ± 0.048 vs. −1.076 ± 0.424, *p* = 0.015).

### 2.3. Emergence of Resistance

No bacterial subpopulations resistant to daptomycin or ceftobiprole were detected among biofilm-embedded cells with any treatment (monotherapy or combination) in any strain.

## 3. Discussion

PJIs caused by MRSA are difficult to cure. There is a need to find effective treatments with good antibiofilm activity at clinically relevant concentrations in bone tissue. The use of antibiotics with low odds of developing resistance is critical in the first days after surgical debridement, when the risk of emergence of resistant populations is greater due to a higher bacterial inoculum and the presence of more exponentially growing bacteria [2]. The availability of beta-lactams with intrinsic activity against MRSA offers a therapeutic alternative to conventional treatments based on glycopeptides or lipopeptides. Using a validated in vitro PK/PD biofilm model, we evaluated the comparative anti-biofilm efficacy of ceftobiprole, alone and in combination with daptomycin.

Our results showed that both ceftobiprole and daptomycin tested at concentrations expected in bone tissue were active as monotherapies against 48-h, mature biofilms of two clinical MRSA strains. The superiority of one regimen over the other was not demonstrated. Daptomycin showed notable inter-strain variability, with a log_10_ CFU/cm^2^ range of −0.56 to −1.50, consistent with values reported in other studies using the CBR and strains of comparable susceptibility [14]. The activity of ceftobiprole was more reproducible in the strains tested, with a mean of −1.49 log_10_ CFU/cm^2^ reduction at 56 h.

The activity of ceftobiprole against staphylococcal biofilms observed in our study is consistent with previous research. Abbanat et al. showed that a range of ceftobiprole concentrations exhibited activity (CFU reductions of 1.5 log_10_ to ≥2.5 log_10_) against staphylococcal biofilms in two static in vitro models [8]. Vaudaux et al. tested the activity of ceftobiprole in a rat tissue cage model of a 14-day MRSA infection and observed reductions of log_10_ 0.68 ± 0.28 CFU/mL after 7 days of treatment [9], similar to vancomycin. Ceftobiprole’s antimicrobial activity in bone was also evaluated by Yin et al. in a rabbit model of MRSA tibial osteomyelitis, showing 100% bacterial clearance from bone after four weeks of treatment [10], higher than vancomycin and linezolid (73% bacterial clearance each). Of interest, ceftobiprole has already been used in cases of bone and joint infections, including prosthetic joint infections [15].

The combination of daptomycin and beta-lactams has shown in vitro synergism against *S. aureus* strains with different resistance patterns, being particularly active against daptomycin non-susceptible strains [6]. This synergistic effect has also been studied in an animal foreign body infection model [7,16]. In our study, the combination of ceftobiprole and daptomycin increased the activity of the individual monotherapies only in one of the two strains (MRSA-1811), without achieving synergistic activity. The lack of synergy observed contrasts with the results of Barber et al. [11], who showed strong synergy between the two compounds, particularly against less fit VISA strains, although their study was a static in vitro model measuring activity against log-phase bacteria. Another study specifically designed to evaluate the potential synergy of ceftobiprole and 29 different FDA-approved compounds also failed to show synergy in combination with daptomycin [17]. Daptomycin combined with cefazolin also failed to show synergistic activity, and no combination therapy was found to be superior to the other. The comparable activity observed between daptomycin plus ceftobiprole and daptomycin plus cefazolin supports the notion that, under the pharmacokinetic conditions simulated in our model, ceftobiprole—at concentrations expected to be achieved in cortical bone—does not exert a synergistic or even additive effect when combined with daptomycin.

The CBR is a dynamic single-compartment model for studying antimicrobial activity against bacterial biofilms that is able to simulate humanized PK conditions. Barber et al. used it to evaluate the activity of another cephalosporin with intrinsic anti-MRSA activity, ceftaroline, alone and in combination with daptomycin and rifampin against three daptomycin non-susceptible MRSA strains [18]. Their results showed that daptomycin plus ceftaroline was the most efficacious regimen, reaching the bactericidal threshold. Although the high activity of this combination contrasts with our apparently less efficacious results, there were notable differences between the two experiments. First, the pharmacokinetic simulation in Barber et al.’s study reflected the expected *f*C_max_ in plasma [18], whereas we simulated the expected pharmacokinetics in bone. Consequently, our *f*C_max_ was lower than those used in other studies. This primarily penalizes the beta-lactam used, which has a similarly low bone penetration to other cephalosporins [12]. However, we consider that it is important to test antimicrobials at concentrations expected at the site of action, as the results obtained are more readily translatable to the clinic, particularly for bone and joint infections. Second, Barber used daptomycin non-susceptible strains, for which a greater synergistic effect would be expected with the combination regimen. Finally, the biofilm challenged with antibiotics was less mature than ours (40 h vs. 48 h) and biofilms were grown in different materials. All of these may help to explain the differences observed.

Given the pharmacokinetic limitations of systemic antimicrobials in the treatment of PJIs, local antibiotic delivery has long been recognized as an effective strategy, particularly in cases managed with a two-stage prosthesis revision. Recently, the development of resorbable biomaterials, such as calcium sulfate, that serve as antibiotic carriers has enabled their use in more challenging scenarios, including PJIs managed with implant retention [19]. Local antibiotic therapy using these carriers can achieve drug concentrations up to 1000-fold above the MIC and maintain levels exceeding the MIC for 3–4 weeks [20], offering enhanced antibiofilm activity. In addition, the adjunctive use of non-antibiotic approaches, such as the local administration of bacteriophages, has shown promising efficacy in the treatment of PJIs caused by multidrug-resistant organisms [21,22].

Our study has several limitations. First, the CBR has the inherent constraints of an in vitro model, which is unable to reproduce a bacterial biofilm with the same characteristics that would be found in a human infection, particularly PJIs. Nor does this model take into account the effects of the immune system and other host-pathogen interactions (e.g., intracellular infection) on the efficacy of treatment. Nevertheless, it is a validated model that allows for the study of a reproducible biofilm problem under controlled pharmacokinetic conditions. Second, the *f*C_max_ used in the experiments represents the expected cortical bone concentrations, which are typically lower than cancellous bone for beta-lactams, thus posing a significant challenge to the antimicrobials used. The use of low concentrations was intended to simulate a worst-case scenario, but we cannot rule out greater antimicrobial activity in clinical settings of PJI, where drug concentrations may be higher (cancellous bone, periprosthetic soft tissues, synovium, etc.), especially given the lack of consensus in the literature regarding which tissue concentration is most representative of the PJI microenvironment. Our model offers an illustrative stress test for the antimicrobials used and adds relevant new information that should be interpreted with caution by clinicians. Third, it should be noted that the combination was tested in only two MRSA strains with similar resistance patterns to daptomycin; therefore, the possibility of observing a more pronounced effect in daptomycin non-susceptible strains cannot be excluded. However, both were clinical isolates from PJI cases, with MICs spanning a representative range of ceftobiprole susceptibility. In addition, genetic analyses of virulence factors and biofilm-forming ability were not performed, which may limit the interpretation of strain-dependent variability in the treatment response. Fourth, complementary imaging techniques were not employed to assess the actual penetration of antimicrobials into the established bacterial biofilm. Fifth, as our study focused on the immediate postoperative period, rifampicin was not evaluated due to concerns related to its early use. Similarly, other anti-MRSA agents, such as vancomycin, fosfomycin, or ceftaroline, were not included. Finally, the duration of treatment in this validated in vitro model is limited to 56 h; therefore, the possibility of different long-term activity of the tested antimicrobial regimens cannot be ruled out.

## 4. Materials and Methods

### 4.1. Bacterial Strains

Two clinical MRSA isolates (MRSA-1811, from a patient with acute early post-surgical infection and good evolution; and MRSA-1733, from a patient with a chronic infection and bad prognosis) recovered from patients treated at University Hospital 12 Octubre were evaluated in this study. All isolates were stored in cryovials at −80 °C.

### 4.2. Ethical Approval

This study was approved by the research ethics committee of our institution, Hospital 12 Octubre, Madrid, Spain (reference 20/338, approved on 14 July 2020).

### 4.3. Antimicrobials and Susceptibility Testing

Daptomycin (Fisher Scientific, Madrid, Spain), ceftobiprole powder (Advanz Pharma, Geneva, Switzerland), and cefazolin (Sigma–Aldrich, Madrid, Spain) were resuspended in dimethyl sulfoxide (DMSO) plus glacial acetic acid, distilled water, and phosphate buffer solution (PBS), respectively, as previously described [9,23]. For each antibiotic, a stock solution was prepared prior to each experiment, sterilized by filtration through a 0.22 µm cellulose acetate syringe filter (Millipore, Madrid, Spain) and stored throughout the experiment at −20 °C for ceftobiprole and daptomycin, and at 4 °C for cefazolin.

Minimal inhibitory concentrations (MICs) were determined by an E-test (bioMérieux, Madrid, Spain) [23]. Minimum biofilm eradication concentrations (MBECs) were determined using the MBEC assay (Calgary Biofilm Device, Innovotech, Edmonton, AB, Canada) [24].

### 4.4. Dynamic In Vitro Biofilm Model

The CDC biofilm reactor (CBR, Biosurface Technologies Corp., Bozeman, MT, USA) was used according to a previously described protocol [25]. Prior to each experiment, the strain was recovered by two sequential cultures on blood agar, followed by an overnight culture in ten milliliters of tryptic soy broth (TSB, Sigma–Aldrich) at 37 °C and with continuous shaking at 225 rpm. This was followed by another subculture for two hours. Seven milliliters of this early log bacterial suspension was inoculated into the reactor. Biofilms were grown on 24 titanium alloy coupons (TiAlV, diameter: 1.27 cm, total biofilm growth area: 2.53 cm^2^) and conditioned for 48 h: 24 h of static culture in TSB supplemented with 1% D-glucose (Sigma–Aldrich) followed by 24 h of culture under a continuous flow of 20% TSB supplemented with calcium at 50 µg/mL (Sigma–Aldrich). The infusion rate was 10.5 mL/min to ensure that the bacterial residence time in the reactor was less than the bacterial generation time for each strain (33.4 min for MRSA-1733 and 33.9 min MRSA-1811). This enabled the removal of free-floating bacteria while providing fresh media and nutrients to the biofilm-embedded bacteria. The culture medium was maintained at 37 °C in the reactor and continuously mixed at 130 rpm using a magnetic stirrer to generate shear forces to promote homogenous biofilm formation.

During the treatment phase, antibiotics were administered as bolus dosing to simulate the free peak concentrations (*f*C_max_) expected in cortical bone at standard dosing (10 mg/kg/24 h for daptomycin, 500 mg/8 h for ceftobiprole, and 2 g/8 h for cefazolin) [12,13]. Cefazolin alone was not tested, as no activity was expected in the MRSA strains. The flow rate of calcium-supplemented 20% TSB was then adjusted to simulate the human elimination half-life of each drug [12]. When testing combinations of two antibiotics with different half-lives, additional peristaltic pumps and compartments were added, as described elsewhere [26].

### 4.5. Pharmacokinetic Analysis

For pharmacokinetic (PK) validation, experiments were performed for each antimicrobial regimen in the absence of bacterial inoculum, using saline as the medium. Antibiotics were administered and perfusion pumps calibrated to replicate the target C_max_ and elimination half-life, as previously described. Samples were collected at predefined time points and stored at 4 °C until analysis. Antimicrobial concentrations were determined using an agar diffusion bioassay [27] with Difco Antibiotic Medium No. 1 (Becton Dickinson), employing *Kocuria rhizophila* ATCC 9341 for daptomycin, *Escherichia coli* ATCC 25922 for ceftobiprole, and *Staphylococcus aureus* ATCC 29213 for cefazolin. Agar plates were seeded with the respective indicator strain, and eight serial dilutions of known antibiotic concentrations were applied to cellulose discs. Following 24 h of incubation, inhibition zones were measured using a high-precision digital caliper, and standard curves were constructed by linear regression. Experimental samples were processed identically, and antimicrobial concentrations were calculated by interpolation from the corresponding standard curve. Elimination half-lives and AUCs were calculated using the linear trapezoidal method.

### 4.6. Pharmacodynamic Analysis

Three coupons were aseptically collected from the reactor at 0, 4, 8, 24, 32, 48, and 56 h, rinsed twice with sterile saline (SS, 0.9% NaCl) to remove planktonic bacteria, then placed in sterile tubes containing 10 mL of SS. Biofilm-embedded bacteria were recovered by three alternating 1-min cycles of vortexing and sonication (100 W–40 KHz; LT-100 PRO; Tierratech, Cantabria, Spain) and a final minute of vortexing. The samples were serially diluted and plated on tryptic soy agar (TSA) containing 5% sheep blood and incubated at 37 °C for 24 h. Plating was performed with a single streak in the center of the plate and allowed to absorb into the agar before spreading the inoculum to prevent antibiotic carry-over from the sample.

Bacterial counts were expressed as log_10_ CFU/cm^2^. The lower detection limit was 1.597 log_10_ CFU/cm^2^. The log change method was used to evaluate the efficacy of the antimicrobials from time 0 to each time point (Δlog_10_ CFU/cm^2^ = log_10_ CFU/cm^2^ at time t − log_10_ CFU/cm^2^ at time 0 [mean ± SD]), and time–kill curves were plotted. An antimicrobial regimen was considered bactericidal if the reduction was greater than 3 log_10_ CFU/cm^2^. Combinations were considered additive or synergistic when bacterial killing was greater than 1 and 2 log_10_ CFU/cm^2^, respectively, compared to the best monotherapy. Antagonism was considered when the combination reduced bacterial killing by >1 log_10_ CFU/cm^2^ compared to the monotherapy.

### 4.7. Detection of Resistance Development

Throughout all experiments, screening was performed for the emergence of bacterial subpopulations resistant to each antibiotic regimen. For each antibiotic, specific agar plates with daptomycin and ceftobiprole concentrations of 1 mg/L and 2 mg/L, respectively, were prepared prior to each experiment and stored at 4 °C for a maximum of 72 h until use.

### 4.8. Statistical Analysis

Statistical analyses and graphs were performed using GraphPad Prism version 8 (GraphPad Software, San Diego, CA, USA). The Shapiro–Wilk test was used to test for normality. Differences between groups were compared using the unpaired Student’s *t*-test or Mann–Whitney U test, as appropriate. All tests were two-tailed. *p* values < 0.05 were considered significant.

## 5. Conclusions

At clinically relevant concentrations in bone tissue, ceftobiprole demonstrated similar bacteriostatic activity to daptomycin against MRSA biofilms. The combination of ceftobiprole plus daptomycin improved the activity of each monotherapy only in one of the strains, highlighting inter-strain variability. No combination therapy outperformed the other in either strain.

## Figures and Tables

**Table 1:** Minimum inhibitory concentration (MIC) and minimum biofilm eradication concentration (MBEC) for the two clinical methicillin-resistant *Staphylococcus aureus* (MRSA) studied.

Strain	MIC (mg/L)			MBEC (mg/L)	
	Ceftobiprole	Daptomycin	Cefazolin	Ceftobiprole	Daptomycin
MRSA-1811	1.5	0.5	>32	>512	>256
MRSA-1733	0.5	1	>32	>256	>256

**Table 2:** Pharmacokinetic parameters of the antibiotics used in the in vitro pharmacokinetic/pharmacodynamic model.

Antibiotic (Usual Human Dosage)	*f*C_max_ Plasma ^a^ (mg/L)	Bone Penetration ^b^ (%)	*f*C_max_ Model (mg/L)			Half-Life (t_1/2_) (h)			*f*AUC_0–24 h_ (mg·h/L)
			Theoretical	Experimental	Error (%)	Theoretical	Experimental	Error (%)	
Ceftobiprole (500 mg/8 h)	42.5	21.4	9.1	8.9	−1.7	3	2.89	−3.5	40.42
Daptomycin (10 mg/kg/24 h)	141.1	8.6	12.1	11.2	−7.4	8	7.66	−4.2	114.7
Cefazolin (2 g/24 h)	404	17.9	72.3	77.0	6.6	1.8	1.86	3.1	224.6

^a^ According to Grayson et al. [12]. ^b^ According to Landersdorfer et al. [13].

## Data Availability

Data is contained within the article or Supplementary Materials.

## Supplementary Materials

The following supporting information can be downloaded at: https://www.mdpi.com/article/10.3390/antibiotics14040386/s1, Figure S1: Pharmacokinetic assessment of experimental antimicrobial regimens by bioassay.
