# Supplementary material for: Efficacy of Ceftobiprole and Daptomycin at Bone Concentrations Against Methicillin-Resistant Staphylococcus aureus Biofilm: Results of a Dynamic In Vitro PK/PD Model
Source: Antibiotics (Basel). 2025 Apr 5;14(4):386. doi: 10.3390/antibiotics14040386 (PMC12024218; doi:10.3390/antibiotics14040386)
Supplement: Supplementary file 1 [file antibiotics-14-00386-s001.zip › antibiotics-3547651-supplementary.pdf]

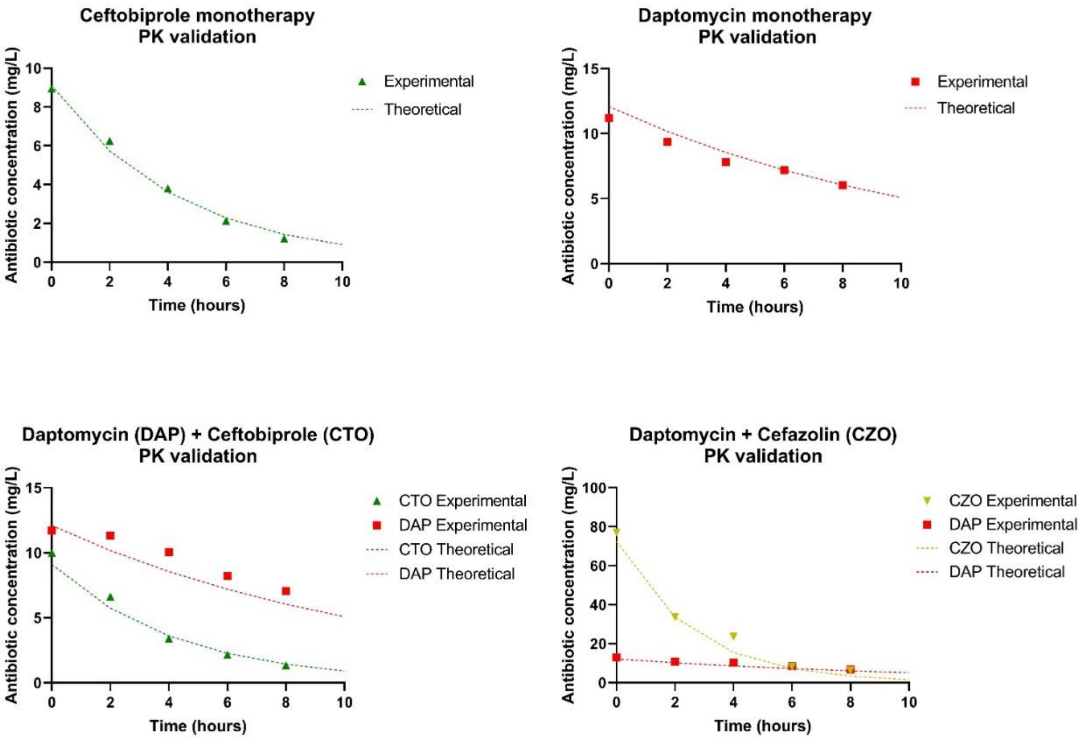

Supplementary Figure S1. Pharmacokinetic assessment of experimental antimicrobial regimens by bioassay.
